# Forsythiaside B ameliorates coagulopathies in a rat model of sepsis through inhibition of the formation of PAD4-dependent neutrophil extracellular traps

**DOI:** 10.3389/fphar.2022.1022985

**Published:** 2022-11-02

**Authors:** Wenju He, Qiang Xi, Huantian Cui, Pingping Zhang, Rui Huang, Taihuan Wang, Dongqiang Wang

**Affiliations:** ^1^ Department of Integration of Traditional Chinese and Western Medicine, First Central Hospital Affiliated to Nankai University, Tianjin First Central Hospital, Tianjin, China; ^2^ Department of Practice and Education, Tianjin University of Traditional Chinese Medicine, Tianjin, China; ^3^ Shandong Provincial Key Laboratory of Animal Cell and Developmental Biology, School of Life Sciences, Shandong University, Qingdao, China; ^4^ Department of Graduate School, Tianjin University of Traditional Chinese Medicine, Tianjin, China

**Keywords:** forsythiaside B, sepsis, coagulation disorders, neutrophil extracellular trap, PAD4

## Abstract

Forsythiaside B (FTB) is one of the main components of *Forsythia suspensa* (Thunb.) Vahl and exerts anti-inflammatory and anti-oxidative effects. However, its mechanism of action as a treatment for sepsis remains unclear. In this study, we developed a rat model of sepsis using cecal ligation and puncture (CLP) to investigate the effects of FTB on sepsis-associated coagulopathies. Using rats with sepsis, we investigated the effects of FTB on neutrophil extracellular trap (NETs) formation and peptidylarginine deiminase 4 (PAD4) expression in neutrophils. NET (DNase1) and PAD4 (Cl-amidine) inhibitors were used to further investigate whether FTB mitigates sepsis-associated coagulopathies by inhibiting PAD4-dependent NETs production. Our results showed that treatment with FTB increased the survival rate, ameliorated the CLP-induced inflammatory response and multiple organ dysfunction, and reduced CLP-induced pathological changes. FTB also alleviated the associated coagulopathies. Additionally, we demonstrated that treatment with FTB inhibited NETs formation and downregulated PAD4 expression in peripheral neutrophils. The effects of FTB on coagulopathies were similar to those of monotherapy with NET or PAD4 inhibitors. In conclusion, our study confirmed that FTB can alleviate coagulopathies in rats with sepsis. The underlying mechanism of FTB’s effect consists in inhibition of PAD4-dependent NETs formation.

## Introduction

Sepsis is a systemic inflammatory response syndrome that can be accompanied by organ dysfunction or even shock, and, in severe cases, it results in death (Russell, 2006). Coagulopathies that occur in sepsis range from a small decrease in platelet (PLT) count and subclinical prolongation of global clotting time to fulminant disseminated intravascular coagulation (DIC). Patients with sepsis-associated DIC may have a manifest thromboembolic disease or clinically less apparent microvascular fibrin deposition, which mainly manifests as multiple organ dysfunction (Schultz et al., 2013).

Recently discovered neutrophil extracellular traps (NETs) represent a new neutrophil-related extracellular mechanism for killing pathogens. NETs are reticular fiber structures formed by the release of intracellular components of neutrophils to the extracellular space. They are produced following the activation of neutrophils owing to various p imilarly, neutrophils activated during sepsis can limit, trap, and kill pathogens by releasing NETs. However, abnormally increased levels of NETs lead to the development of thrombosis by damaging endothelial cells, activating PLTs, and releasing tissue factor (TF), further resulting in coagulopathies and aggravating organ dysfunction (McDonald et al., 2017). Therefore, inhibition of NET formation can alleviate coagulopathies and delay the development of sepsis-related DIC.

Traditional Chinese medicines (TCM) offer distinct advantages in the treatment of sepsis. Liu-Shen-Wan protects against sepsis by lowering plasma levels of tumor necrosis factor-α (TNF-α) and malondialdehyde (MDA) and enhancing the phagocytic capability of peritoneal macrophages (Ma et al., 2006). *Rheum palmatum* L. can effectively reduce gastrointestinal, pulmonary, and hepatic damage caused by sepsis, possibly owing to mechanisms such as counteracting oxidative stress and inflammation, improving microcirculatory disorders, and maintaining immune homeostasis (Lai et al., 2015). In rats with cecal ligation and puncture (CLP)-induced sepsis, injection with Xue-Bi-Jing can reduce the serum levels of pro-inflammatory factors (Jiang et al., 2013).

Forsythiaside B (FTB) is one of the main ingredients of the TCM *Forsythia suspensa* (Thunb.) Vahl and exerts anti-bacterial and antioxidant effects (Nazemiyeh et al., 2007; Cai et al., 2014). FTB can prevent lipopolysaccharide (LPS)-induced acute lung injury by reducing inflammatory cell infiltration and inhibiting toll-like receptor 4 (TLR4)/NF-κB signaling pathways (Liu et al., 2019). However, the mechanism of action of FTB in the treatment of sepsis remains unclear. In this study, we first developed a rat model of sepsis using CLP to evaluate the effects of FTB on associated coagulopathies. Additionally, we investigated the effects of FTB on NETs formation and peptidylarginine deiminase 4 (PAD4) expression in neutrophils isolated from rats with sepsis. Furthermore, these rats were treated with NETs and PAD4 inhibitors to investigate whether FTB alleviates coagulopathies by inhibiting PAD4-dependent NETs production.

## Experimental methods

### Experimental animals and reagents

A total of 360 male Sprague-Dawley rats, weighing 200 ± 20 g, were purchased from Beijing Huafukang Bioscience Co., Ltd. (Beijing, China) and left to acclimatize for 1 week, in a quiet environment at 20 °C and relative humidity of 55%–60%. They had *ad libitum* access to adequate clean water and food. This study was approved by the Ethics Committee of Tianjin First Central Hospital.

FTB (B20728) was purchased from Shanghai Yuanye Bio-Technology Co., Ltd. (Shanghai, China). Deoxyribonuclease I (DNase1, D8071), rat blood neutrophil isolation kit (P9200), and 4′,6-diamidino-2-phenylindole (DAPI, C0060) were obtained from Beijing Solarbio Science and Technology Co., Ltd. (Beijing, China). Cl-amidine (GC11032) was purchased from GlpBio Technology LLC (United States). Alanine transaminase (ALT, C009-2-1), aspartate transaminase (AST, C010-2-1), creatinine (Cr, C011-2-1), and blood urea nitrogen (BUN, C013-2-1) test kits were purchased from the Nanjing Jiancheng Biological Engineering Institute (Nanjing, China). Rat interleukin (IL)-1β (EK301B), IL-6 (EK306), TNF-α (EK382) enzyme-linked immunosorbent assay (ELISA) kits were purchased from Multi Science Biotechnology Co. Ltd. (Hangzhou, China). Rat von Willebrand factor (vWF, ml003160), thrombomodulin (TM, ml059145), p-selectin (ml102808), plasminogen activator inhibitor 1 (PAI-1, ml003024), platelet-activating factor (PAF, ml003008), thrombin–antithrombin complex (ml003154), and C-reactive protein (CRP, ml038253) ELISA kits were purchased from Shanghai Enzyme-linked Biotechnology Co. Ltd. (Shanghai, China). Rabbit antithrombin (bs-1914R) was obtained from Beijing Biosynthesis Biotechnology Co., Ltd. Rabbit polyclonal antibodies against citrullinated histone H3 (Cit H3) (ab5103) were obtained from Abcam (Shanghai, China). Anti-myeloperoxidase (MPO) monoclonal antibodies (GB12224) were purchased from Servicebio Co., Ltd. (Wuhan, China). Anti-PAD4 polyclonal antibodies (17373-1-AP) and anti-β-actin monoclonal antibodies (66009-1-Ig) were obtained from Proteintech (Wuhan, China).

### Development of the rat model of sepsis

A rat model of sepsis was developed using the CLP method (Dejager et al., 2011). Rats were fasted for 12 h preoperatively. After anesthesia with isoflurane, the skin in the middle of the anterior abdomen was prepared and disinfected. Subsequently, a 2 cm incision was made in the midline of the abdomen. The ileocecum was located, the cecum was isolated and exteriorized, and the mesentery and cecum were separated. The cecum was ligated at its end 5 mm from the ileocecum using 3–0 sutures. Two perforated punctures were made in the intestinal wall on both sides at 0.8 and 1.0 cm, respectively from the root of the appendix and the distal end of the ligature, using a syringe needle (20 G). A small amount of intestinal content was excreted, and a 3 × 0.5 × 30 mm drain was placed in the perforated punctures. Thereafter, the cecum was placed back into the abdominal cavity and the incision was sutured and disinfected.

### Experimental groups and dosing regimens

**FIGURE 1:**
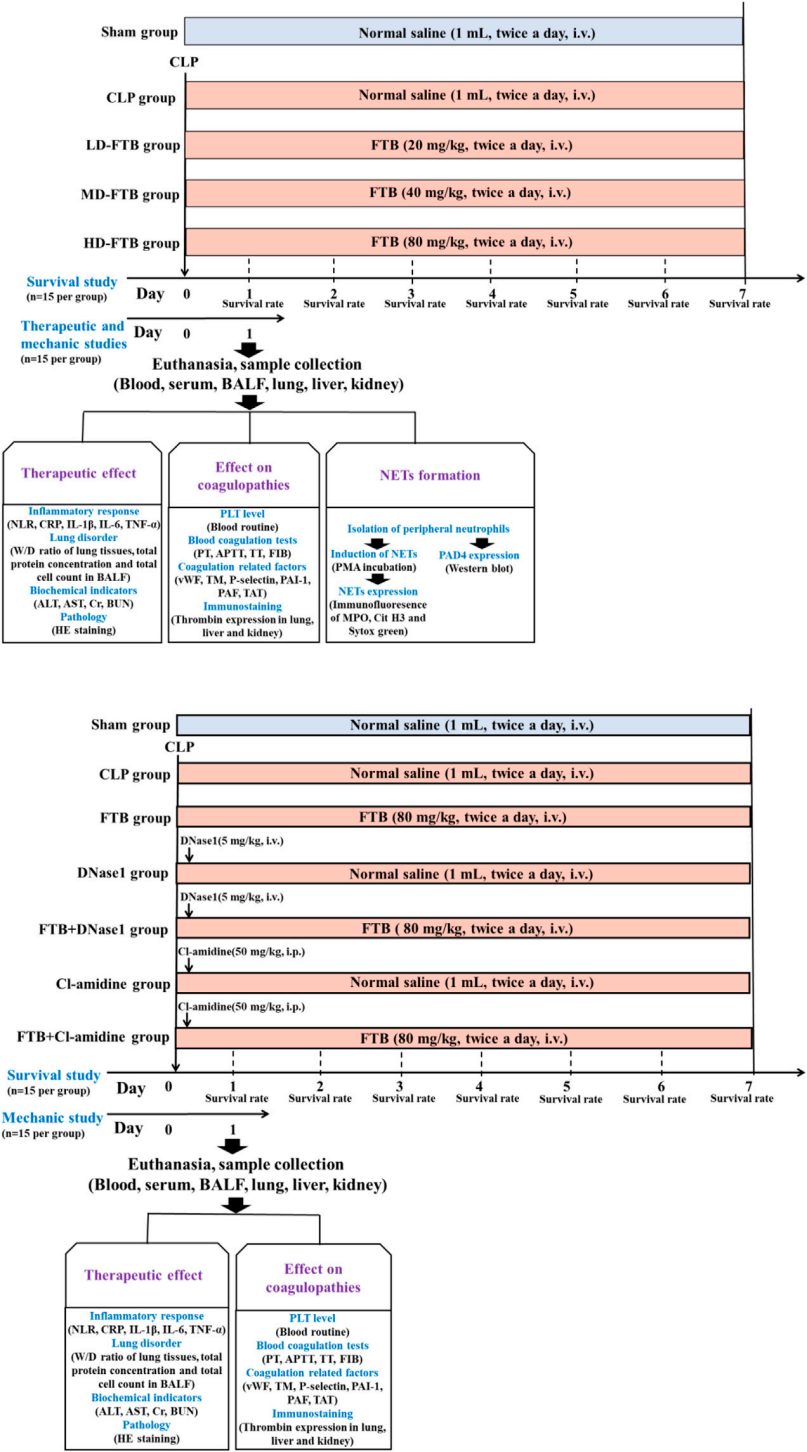
Overview of the experimental design of the study assessing the therapeutic effects of forsythiaside B (FTB) on sepsis **(A)** and of the study assessing the involvement of neutrophil extracellular traps (NETs) and peptidylarginine deiminase 4 (PAD4) in the effect of FTB using corresponding inhibitors **(B)**. **(A)** Rats were subjected to cecal ligation and puncture (CLP) to induce sepsis and were treated with different dosages of FTB; the survival rate was calculated for 7 days after CLP (n = 15 per group). The effects of FTB on the markers of the disease and NETs formation were investigated 24h after CLP (n = 15 per group). **(B)** Septic rats received NETs (DNase1) and PAD4 (Cl-amidine) inhibitors followed by treatment with FTB. The survival rate (n = 15 per group) and the effects of FTB on sepsis and coagulation function (n = 15 per group) were studied.

To study the effects of FTB on coagulation function and NETs formation in rats with sepsis, 150 rats were selected and randomly divided into sham, CLP, CLP + low-dose FTB (LD-FTB), CLP + medium-dose FTB (MD-FTB), and CLP + high-dose FTB (HD-FTB) groups, with 30 rats in each group. In rats in the sham group, the abdominal wall was incised and sutured without CLP. After surgery, rats in the LD-FTB, MD-FTB, and HD-FTB groups received intravenously FTB 20 mg/kg, 40 mg/kg, and 80 mg/kg, respectively, and those in the sham and CLP groups received intravenously the same volume of normal saline. Drugs were administered once every 12 h (Figure 1A).

In a second study, investigating the effect of NETs (DNase1) and PAD4 (Cl-amidine) inhibitors on FTB, 210 rats were selected and randomly divided into sham, CLP, FTB, DNase1, FTB + DNase1, Cl-amidine, and FTB + Cl-amidine groups, with 30 rats in each group. CLP was performed to induce sepsis in all rats, except those in the sham group. For rats in the sham group, the abdominal wall was incised and sutured without performing CLP. After surgery, animals received the following: FTB group, FTB 80 mg/kg intravenously; DNase1 group, DNase1 5 mg/kg, one injection into the caudal vein 1 h after the model was established; FTB + DNase1 group, DNase1 5 mg/kg as previously described + FTB 80 mg/kg intravenously; Cl-amidine group, Cl-amidine 50 mg/kg, one intraperitoneal administration 1 h after the model generation; FTB + Cl-amidine group, Cl-amidine as previously described + FTB 80 mg/kg intravenously; the same volume of normal saline was intravenously injected into rats in the Sham and CLP groups. FTB and normal saline were administered once every 12 h (Figure 1B).

Fifteen rats from each group were monitored following CLP to measure the survival rates. The number of surviving rats in each group was counted every day for 7 days. The following formula was used to assess survival: Survival (%) = number of surviving rats× 100/15, and the survival curve was drawn based on the daily number of surviving rats. The other 15 rats in each group were anesthetized with pentobarbital sodium *via* intraperitoneal injection (50 mg/kg) at 24 h after model generation, and their blood, bronchoalveolar lavage fluid (BALF), and lung, liver, and kidney tissues were collected for subsequent tests.

### Assessment of PLT and coagulation function

Twenty-four hours after drug administration and CLP induction, the rats were anesthetized. The abdominal cavity was cut open and blood was collected from the abdominal aorta. A total of 200 μL of the collected blood was gently mixed in an EDTA-K2 anticoagulant vacuum blood collection tube and placed at room temperature for 15 min, and the PLT count and neutrophil/lymphocyte ratio (NLR) in the peripheral blood were evaluated using an automatic hemocytometer analyzer. Subsequently, 200 μL of blood was collected again and gently mixed in a sodium citrate anticoagulation vacuum blood collection tube. Prothrombin time (PT), activated partial thromboplastin time (APTT), thrombin time (TT), and fibrinogen (FIB) levels were determined using an automatic coagulation analyzer. These tests were completed within 2 h after blood collection.

### Calculation of Wet-to-Dry (W/D) ratio of lung tissues and measurement of total protein concentration and total cell count in BALF

The W/D ratio of lung tissues, total protein concentration, and total cell count in BALF were determined as previously described (He et al., 2022) to evaluate the effect of FTB on sepsis-induced lung injury in rats.

### Serological testing of liver and renal function

Blood taken from the rats was placed at room temperature and centrifuged for 10 min at 3,000 rpm, and the serum was further collected. The activities of serum liver function-related indices (ALT and AST) and the levels of serum renal function-related indices (Cr and BUN) were tested according to the manufacturer’s instructions (Nanjing, China).

### Enzyme-linked immunosorbent assay (ELISA)

Serum inflammatory factors (IL-1β, IL-6, TNF-α, and CRP) and coagulation factors (vWF, TM, P-selectin, PAI-1, PAF, and TAT) were measured according to the manufacturer’s instructions.

### Hematoxylin and eosin (HE) staining

After the rats were sacrificed, their lung, liver, and kidney tissues were collected, fixed in formalin solution, embedded in paraffin, cut into 3 μm sections, stained with routine HE staining, and sealed with neutral gum. Histopathological changes in the rats from each group were observed under a light microscope. Tissue injury scores in the lung (Matute-Bello et al., 2011), liver (Hart, N. et al., 2004) and kidney (Liang et al., 2020) were evaluated based on previously published methods.

### Immunohistochemistry (IHC)

The lung, liver, and kidney tissues of the rats were collected, fixed in formalin solution, embedded in paraffin, cut into 3 μm sections, dewaxed, and hydrated for antigen retrieval. The samples were permeabilized with 0.5% Triton X-100 for 5 min, sealed with 5% bovine serum albumin (BSA), and incubated at 37°C for 1 h. The samples were incubated with the primary antibody, anti-thrombin (1:500), overnight at 4°C, washed three times with phosphate-buffered saline (PBS), and a secondary antibody was added, followed by incubation at room temperature for 1 h and washing three times with PBS. Thereafter, the samples were subjected to routine 3,3′-diaminobenzidine (DAB) staining, counterstaining, dehydration, clearance, and the slides were sealed with neutral balsam. The positive expression area was quantified using Image-Pro Plus 6.0, and the average optical density (AOD) was calculated using the following formula:

AOD=integrated optical density (IOD)/total area

### Neutrophil extraction

Twenty-four hours after drug administration and CLP induction, 5 ml of peripheral blood was collected from the rats in each group and placed in a heparin sodium anticoagulant vacuum collection tube. Neutrophils were isolated using animal peripheral blood neutrophil isolation kits. Specific steps were performed according to the manufacturer’s instructions: 4 ml of reagent A was added to the collected anticoagulant, 2 ml of reagent C was carefully layered onto reagent A to form a gradient interface, and the blood was laid flat above the surface of the separation medium. We paid attention to keep the interface between the two liquid surfaces clear. The samples were centrifuged for 20–30 min at room temperature with a centrifugal force of 500–1,000 × *g*. After centrifugation, two layers of annular milky white cells were observed in the centrifuge tube; the upper layer consisted of mononuclear cells and the lower layer of neutrophils. The neutrophil layer was carefully pipetted into a clean (15 ml) centrifuge tube, 10 ml of PBS were added, the samples were centrifuged for 10 min at a centrifugal force of 250 × *g*, and the precipitate was collected. The cells were resuspended in 5 ml PBS, the samples were centrifuged for 10 min with a centrifugal force of 250 x g, and the obtained precipitate consisted of neutrophils.

### NET induction and immunofluorescence testing for NET levels

The purified neutrophils were resuspended in a cell culture medium comprising 89% Dulbecco’s modified Eagle medium (DMEM), 10% fetal bovine serum (FBS), and 1% penicillin–streptomycin, and the cells were inoculated in a 6-well plate at a density of 2 × 10^5^ cells/mL and incubated for 1 h. Subsequently, NET formation was induced with phorbol-12-myristate-13-acetate (PMA) (100 mM) for 3 h (Bystrzycka et al., 2017), cells were fixed with 4% paraformaldehyde for 15 min, permeabilized with 0.5% Triton X-100 for 5 min, sealed with 5% BSA, and incubated at 37°C for 1 h. Fluorescent antibodies against Cit H3 (1:200) and MPO (1:1000) were added, and the slides were incubated overnight at 4°C. After washing the slides three times with PBS, the secondary antibody was added, and the plate was incubated at room temperature for 1 h. SYTOX Green nucleic acid stain (5 μM) was added, and the corresponding reaction occurred at room temperature for 15 min. The plate was washed twice with PBS, counterstained with DAPI for 10 min, and washed thrice with PBS. The slides were sealed with an anti-fluorescence quenching agent and an immunofluorescence microscope was used to observe the images. The fluorescence intensity and range were quantified using Image-Pro Plus 6.0, and AOD was calculated.

### Western blot

The extracted neutrophils were placed in ice-cold radioimmunoprecipitation assay lysis buffer and lysed for 30 min on ice. The sample was centrifuged for 10 min at 12,000 rpm and 4°C, and the supernatant was pipetted into a new tube. The protein content of the cell supernatant was determined using bicinchoninic acid protein assay kits to ensure that the protein content of each sample was consistent. Subsequently, 20 μg of each sample were submitted to electrophoresis on 10% sodium dodecyl sulfate-polyacrylamide gel. The target protein isolated from the gel was transferred onto a polyvinylidene fluoride membrane activated with methanol. The membrane was blocked at room temperature with 5% non-fat dry milk for 2 h and then incubated overnight at 4°C with anti-PAD4 (1:3000) and anti-β-actin (1:5000) antibodies. The next day, the membrane was washed three times with Tris-buffered saline containing 2% Tween 20 (TBST) buffer for 10 min each. The corresponding secondary antibodies were added and incubated at room temperature for 2 h. The membrane was washed three times with TBST for 5 min each. The bands were visualized using enhanced chemiluminescence, with β-actin as the housekeeping protein, and images were acquired using a gel imaging analysis system. Additionally, the gray values of each band were analyzed using the ImageJ software, and the relative protein expression of PAD4 was calculated. The following formula was used: Relative protein expression = target protein gray value/housekeeping protein gray value.

### Statistical analysis

The experimental results were analyzed using the SPSS Statistics software version 20.0. The measurement data are expressed as mean ± standard deviation (SD). A *t-test* was performed to compare independent means two by two, and one-way analysis of variance (ANOVA) followed by Scheffe post-hoc test was used to compare means across multiple groups. Statistical significance was set at *p* levels *< 0.05*.

## Results

### Therapeutic effects of FTB on rats with sepsis

**FIGURE 2:**
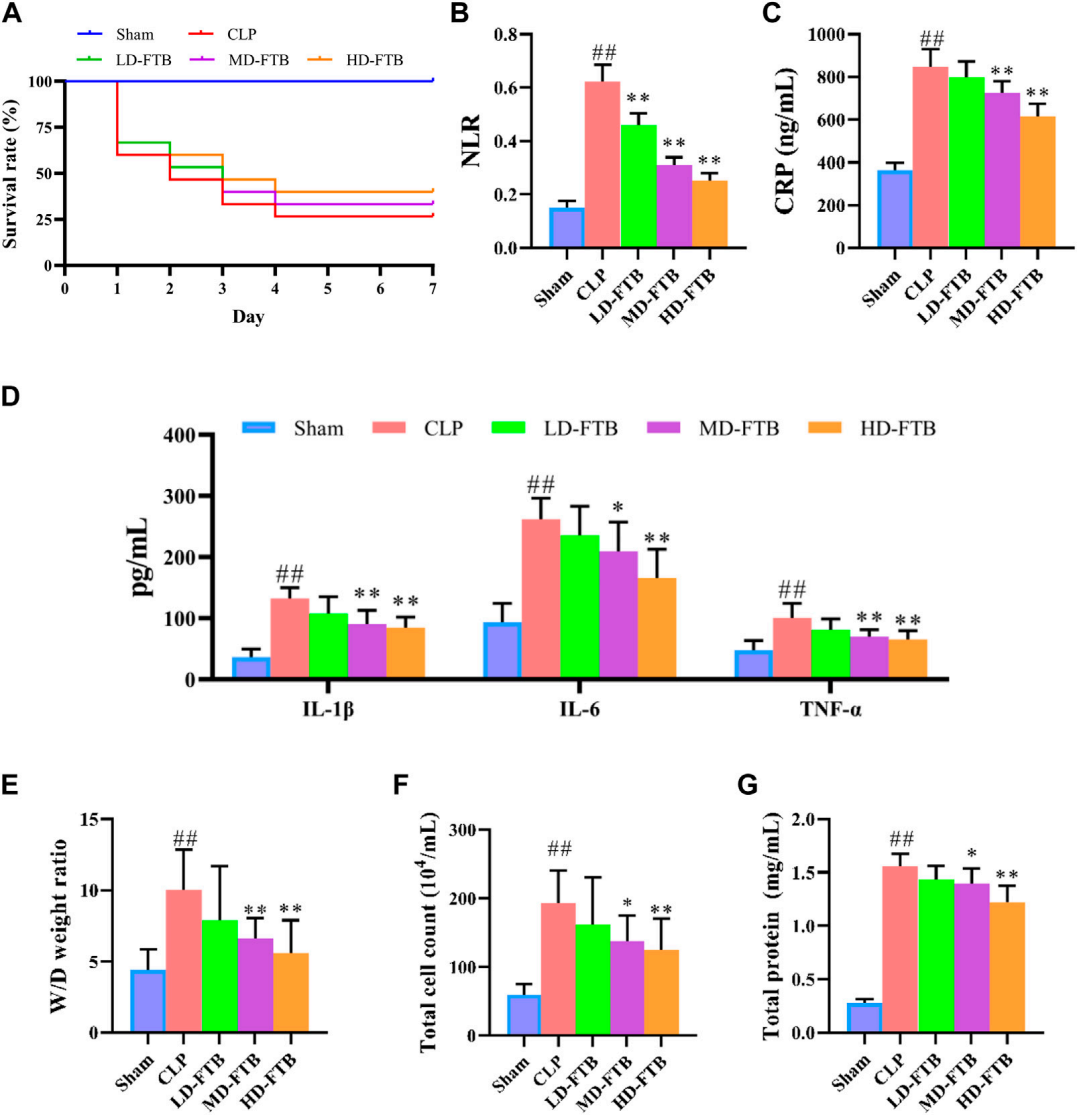
Effects of treatment with FTB on survival rate, inflammatory response and lung permeability in rats with sepsis. **(A)** FTB treatment increased the survival rate in rats with sepsis **(B–D)** The effects of FTB on inflammatory response were observed by testing the neutrophil/lymphocyte ratio (NLR) in peripheral blood **(B)** through blood routine examination and measuring the serum levels of CRP **(C)**, IL-1β **(D)**, IL-6 **(D)**, and TNF-α **(D)** using ELISA. FTB treatment lowered NLR and the levels of CRP, IL-1β, IL-6, and TNF-α in rats with sepsis **(E–G)** The changes in lung permeability were assessed through measuring the wet-to-dry (W/D) ratio of lung tissue **(E)**, total cell count **(F)** and in total protein concentration **(G)** in bronchoalveolar lavage fluid (BALF). FTB treatment decreased the W/D ratio of lung tissues, total protein concentration and total cell count in BALF. **(A)**: *n* = 15 per group at day 0; **(B–G)**: sham group (*n* = 15), CLP group (*n* = 8), LD-FTB (*n* = 9), MD-FTB (*n* = 10), HD-FTB (*n* = 12) groups.

Seven days after drug administration and surgery, the survival rates of the rats in the sham and CLP groups were 100% and 26.7%, respectively. Administration of FTB increased the survival of rats with sepsis, and the survival rates in the LD-FTB, MD-FTB, and HD-FTB groups were 26.7%, 33.3%, and 40%, respectively (Figure 2A).

Additionally, rats in the CLP group exhibited a higher NLR than rats in the sham group, and FTB treatment reduced the NLR in rats with sepsis (Figure 2B). ELISA results showed that serum levels of CRP, IL-6, IL-1β, and TNF-α were significantly higher in the CLP group than in the sham group. FTB treatment lowered the levels of CRP, IL-6, IL-1β, and TNF-α in rats with sepsis (Figures 2C,D).

We also evaluated the effects of FTB on sepsis-associated lung, liver, and kidney disorders in rats. The W/D ratio of the lung tissue, total protein concentration, and total cell count in BALF were higher in the CLP group than in the sham group. Compared with the rats in the CLP group, FTB-treated rats showed a lower W/D ratio of lung tissues, total protein concentration, and total cell count in BALF (Figures 2E–G). Assessment of serum indices of the hepatic function showed that serum levels of ALT and AST were significantly higher in the CLP group than in the sham group. Compared with CLP group, the MD-FTB and HD-FTB groups had lower serum levels of ALT and AST (Table 1). The results of the renal function test showed that serum Cr and BUN levels were significantly higher in the CLP group than in the sham group. Compared with the CLP group, FTB group had significantly lower serum levels of Cr and BUN (Table 1).

**TABLE 1:** Changes in ALT and AST activities, Cr and BUN levels after FTB treatment.

Group	ALT(U/L)	AST(U/L)	Cr(μmol/L)	BUN(mmol/L)
Sham	29.3 ± 14.3	32.7 ± 15.1	29.8 ± 13.4	3.1 ± 0.7
CLP	82.8 ± 39.1^##^	105.5 ± 60.3^##^	107.0 ± 20.9^##^	6.7 ± 2.2^##^
LD-FTB	69.7 ± 32.5	108.4 ± 107.1	88.4 ± 27.3	5.8 ± 2.0
MD-FTB	51.0 ± 15.0^*^	44.7 ± 17.8^*^	76.8 ± 32.7^*^	4.8 ± 1.3^*^
HD-FTB	41.9 ± 14.1^**^	42.0 ± 17.3^**^	65.6 ± 30.7^**^	4.6 ± 1.1^**^

Sham group (*n* = 15), CLP group (*n* = 8), LD-FTB (*n* = 9), MD-FTB (*n* = 10), HD-FTB (*n* = 12); Data are presented as the mean ± SD.^##^: *p* < 0.01 as compared to the Sham group;^*^: *p* < 0.05 as compared to the CLP group;^**^: *p* < 0.01 as compared to the CLP group.

**FIGURE 3:**
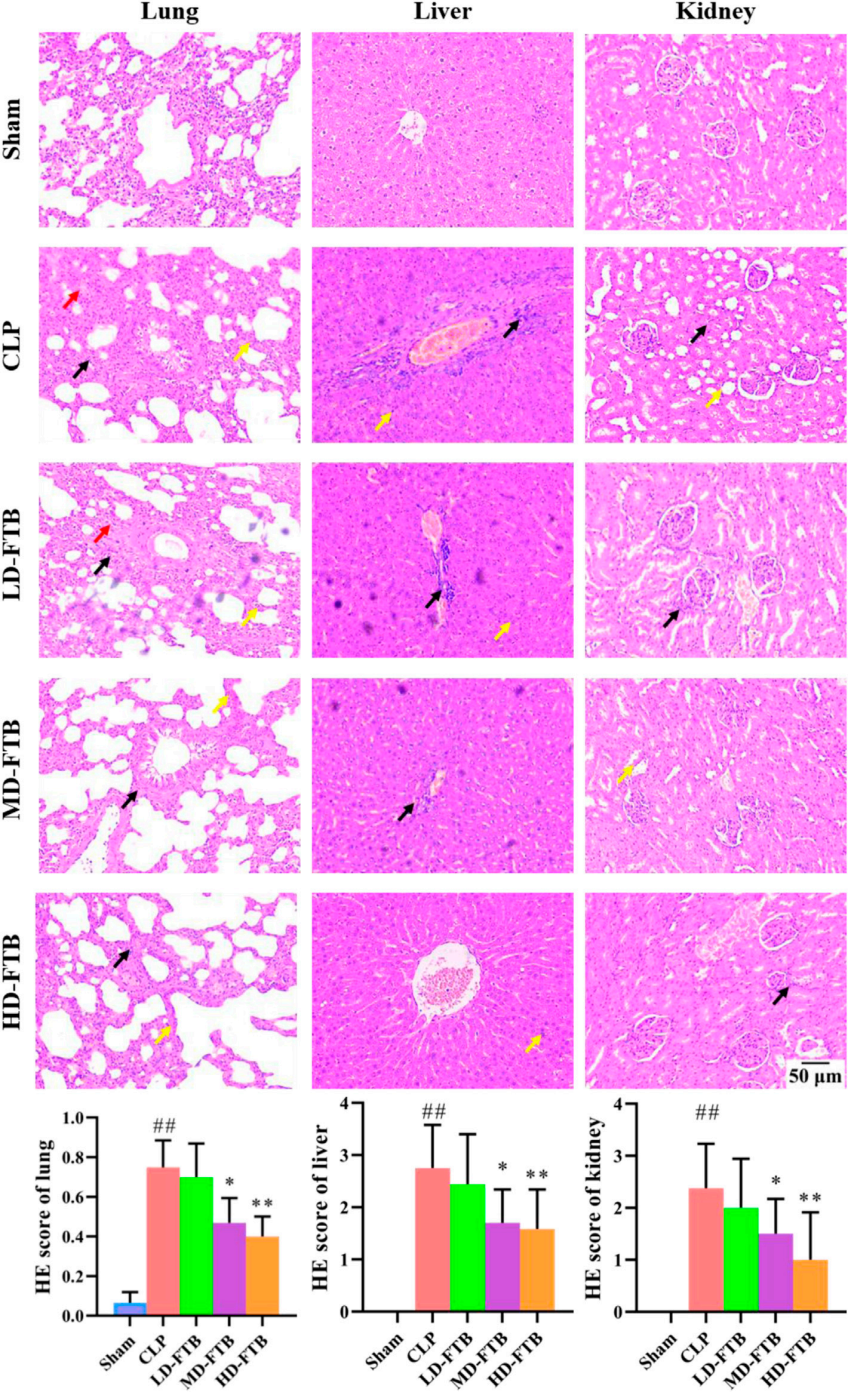
Effects of FTB on CLP-induced pathological changes and tissue injury scores of the lung, liver and kidney in rats. The CLP-induced pathological changes in lung, liver and kidney after treatment with FTB were observed and the tissue injury scores were evaluated by HE staining. Treatment with FTB improved the CLP-induced pathological changes in the lung (red arrows indicate the pulmonary interstitial congestion, yellow arrows indicate the thickened alveolar walls and black arrows indicate the infiltration of inflammatory cells), liver (yellow arrows indicate the necrotic hepatocytes and black arrows indicate the infiltration of inflammatory cells) and kidney (yellow arrows indicate the enlarged lumen of focal tubules and black arrows indicate the interstitial inflammatory cell infiltration), and decreased the tissue injury scores (magnification: ×100). Sham (*n* = 15), CLP (*n* = 8), LD-FTB (*n* = 9), MD-FTB (*n* = 10), HD-FTB (*n* = 12).

HE staining showed that rats in the CLP group had thickened alveolar walls, pulmonary interstitial congestion, inflammatory cell infiltration in the lung; disorganized hepatocytes, disordered hepatic cords, and necrotic hepatocytes, with distinct inflammatory cell infiltration in the liver; focal tubular degeneration and atrophy, enlarged lumen, and interstitial inflammatory cell infiltration in the kidney. Compared with the CLP group, all FTB treatment groups presented an alleviation of the pathological changes, and the improvement was significantly higher in the high-dose group. Similarly, the tissue injury scores of the lung, liver, and kidney were higher in the CLP group than in the sham group, whereas FTB-treated rats showed lower scores than rats in the CLP group (Figure 3).

### Effects of FTB on coagulation function in rats with sepsis

**FIGURE 4:**
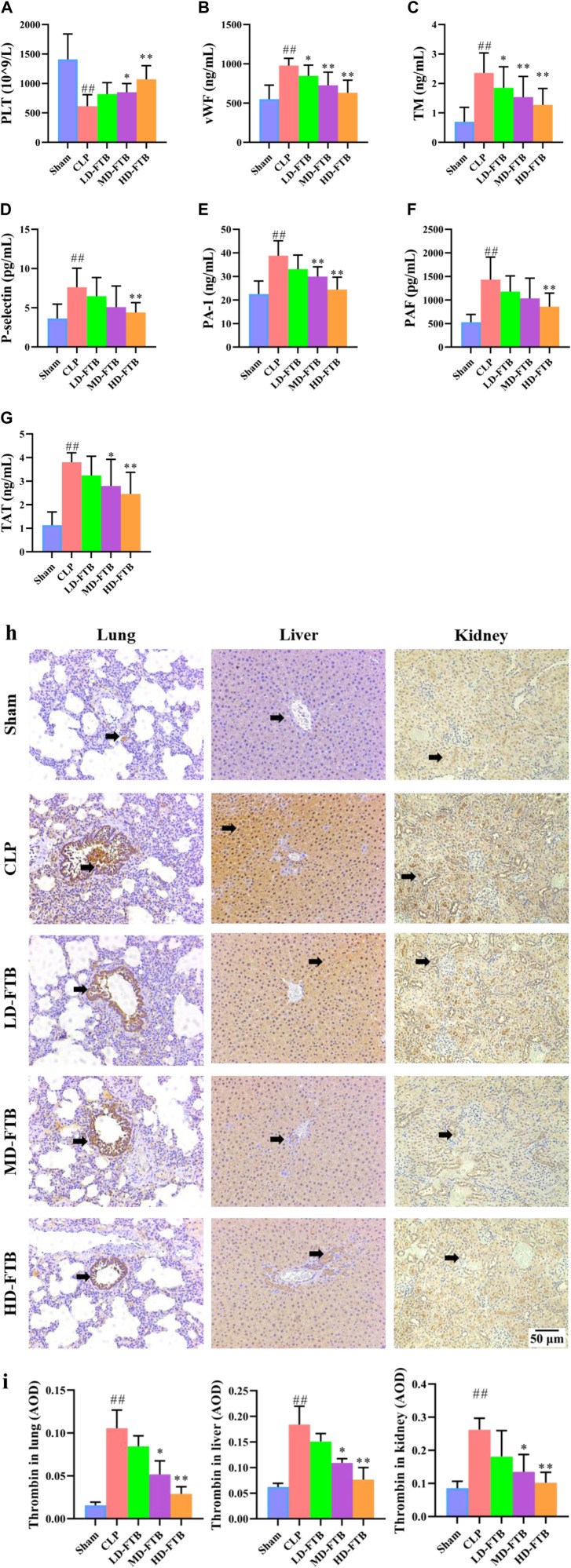
Effects of FTB on coagulation function in rats with sepsis. We investigated the platelet (PLT) count using blood routine examination **(A)**, the serum levels of von Willebrand factor (vWF) **(B)**, thrombomodulin (TM) **(C)**, P-selectin **(D)**, plasminogen activator inhibitor 1 (PAI-1) **(E)**, platelet-activating factor (PAF) **(F)**, and thrombin–antithrombin complex (TAT) **(G)** using ELISA, and the expression of thrombin in lung, liver and kidney using immunohistochemistry (black arrows indicate the positive expression areas) (magnification: ×100) **(H)**; the thrombin-positive expression area detected using immunohistochemistry was quantified based on the average optical density (AOD) **(I)**. FTB treatment increased the PLT count, decreased the levels of coagulation factors (vWF, TM, P-selectin, PAI-1, PAF, TAT) and lowered the expression of thrombin in lung, liver and kidney in rats with sepsis. Sham (n = 15), CLP (*n* = 8), LD-FTB (*n* = 9), MD-FTB (*n* = 10), HD-FTB (*n* = 12).

Compared with the sham group, the CLP group had a significantly lower PLT level, significantly longer APTT and PT and significantly lower FIB levels; compared with the CLP group, the MD-FTB and HD-FTB groups had higher PLT levels blood (Figure 4A), significantly shorter APTT and PT, and significantly higher FIB levels (Table 2).

**TABLE 2:** Changes in APTT, PT, TT and FIB levels after FTB treatment.

Group	APTT(s)	PT(s)	TT(s)	FIB(μg/L)
Sham	15.2 ± 2.1	13.9 ± 2.7	21.8 ± 4.0	3.5 ± 1.0
CLP	51.9 ± 12.0^##^	58.1 ± 13.8^##^	26.1 ± 4.2^#^	2.0 ± 0.1^##^
LD-FTB	37.2 ± 13.3^*^	42.8 ± 22.3	25.2 ± 3.7	2.2 ± 0.7
MD-FTB	33.4 ± 10.3^**^	37.4 ± 5.9^**^	25.0 ± 5.6	2.5 ± 0.5^*^
HD-FTB	27.8 ± 7.4^**^	24.4 ± 5.9^**^	22.9 ± 3.4	3.2 ± 1.0^**^

Sham group (*n* = 15), CLP group (*n* = 8), LD-FTB (*n* = 9), MD-FTB (*n* = 10), HD-FTB (*n* = 12).

ELISA results showed that CLP group had significantly higher levels of serum coagulation factors (vWF, TM, P-selectin, PAI-1, PAF, and TAT) compared with the sham group. Compared with the CLP group, the FTB group had lower levels of serum coagulation factors (vWF, TM, P-selectin, PAI-1, PAF, and TAT) (Figures 4B–G).

The IHC results indicated that the thrombin-positive expression area in the lung, liver, and kidney tissues was significantly higher in the CLP group than in the sham group. Compared with the CLP group, the MD-FTB and HD-FTB groups had smaller thrombin-positive expression areas in the lung, liver, and kidney tissues (Figures 4H,I).

### Effects of FTB on NET formation and PAD4 expression in neutrophils isolated from rats with sepsis

**FIGURE 5:**
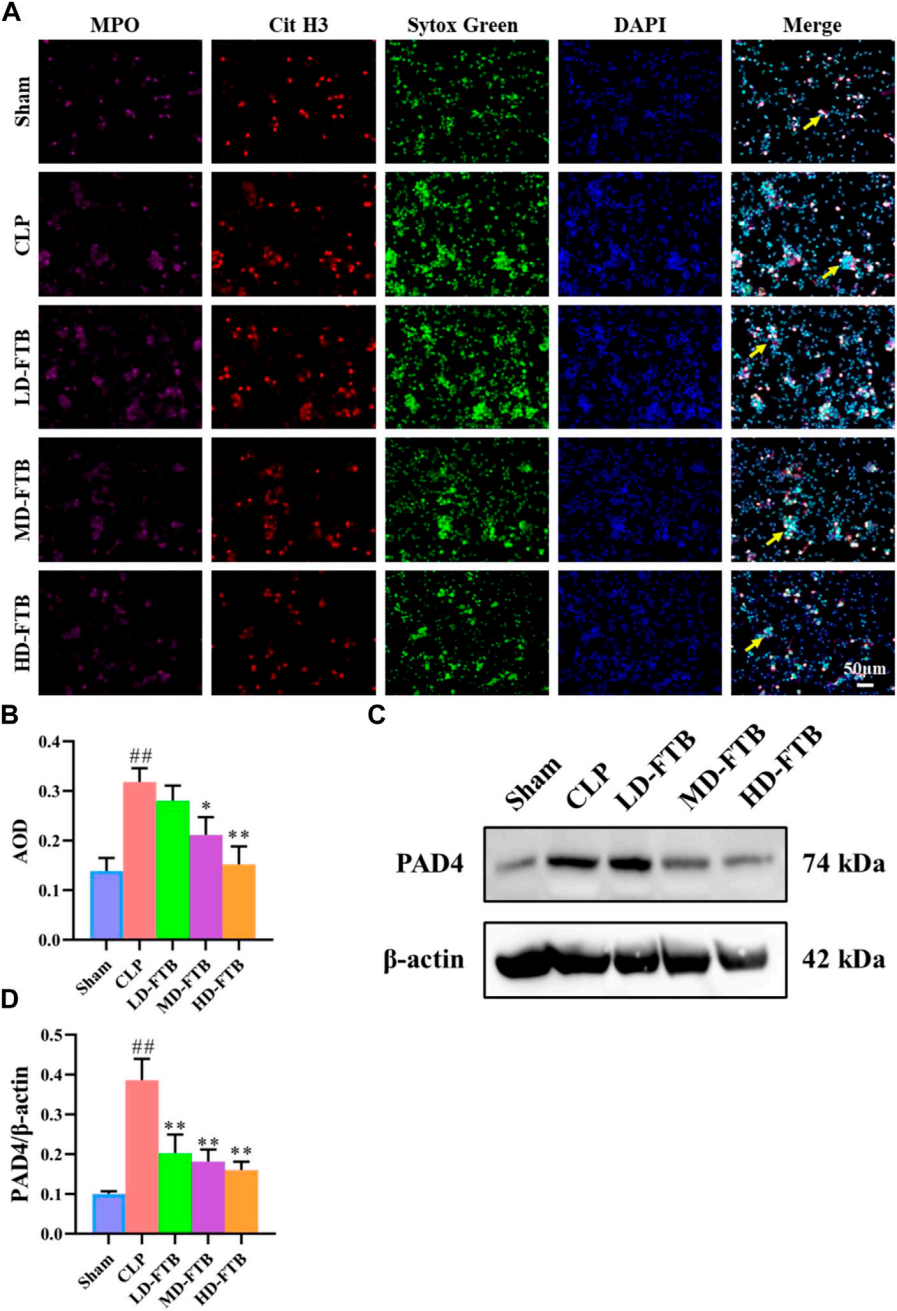
Effects of FTB on NETs formation and PAD4 expression in neutrophils isolated from the peripheral blood of rats with sepsis. The NETs were induced by exposure to phorbol-12-myristate-13-acetate (PMA) (100 mM) for 3 h. The formation of NETs was observed by investigating the expression of myloperoxidase (MPO), citrullinated histone H3 (Cit H3), and Sytox green in cells using immunofluorescence (yellow arrows indicate the positive expression of NETs) **(A)** and the positive expression area was quantified based on AOD **(B)**. The protein expression of PAD4 in neutrophils was measured using Western blot, β-actin was used as a loading control **(C)**. The relative expression of PAD4 was quantified based on the gray values **(D)**. FTB treatment inhibited the NET formation and decreased the PAD4 expression in neutrophils.

Immunofluorescence analysis showed that all the cells expressed MPO and had lobulated nuclei. Furthermore, the Cit H3^+^Sytox Green^+^ fluorescence intensity was significantly higher in the CLP group than in the sham group. Compared with the CLP group, the MD-FTB and HD-FTB groups had significantly lower Cit H3^+^Sytox Green^+^ fluorescence intensity (Figures 5A,B). Results of Western blotting indicated that PAD4 expression in neutrophils was significantly higher in the CLP group than in the sham group. In the LD-FTB, MD-FTB, and HD-FTB groups, drug administration inhibited PAD4 expression in the neutrophils (Figures 5C,D). HD-FTB showed the highest efficacy in reducing the mortality of rats with sepsis, improving organ function, alleviating associated coagulopathies, and inhibiting NET formation. Therefore, the HD-FTB group was selected for the follow-up experiments.

### Therapeutic effects of FTB on rats with sepsis after the inhibition of NET formation and PAD4 activity

**FIGURE 6:**
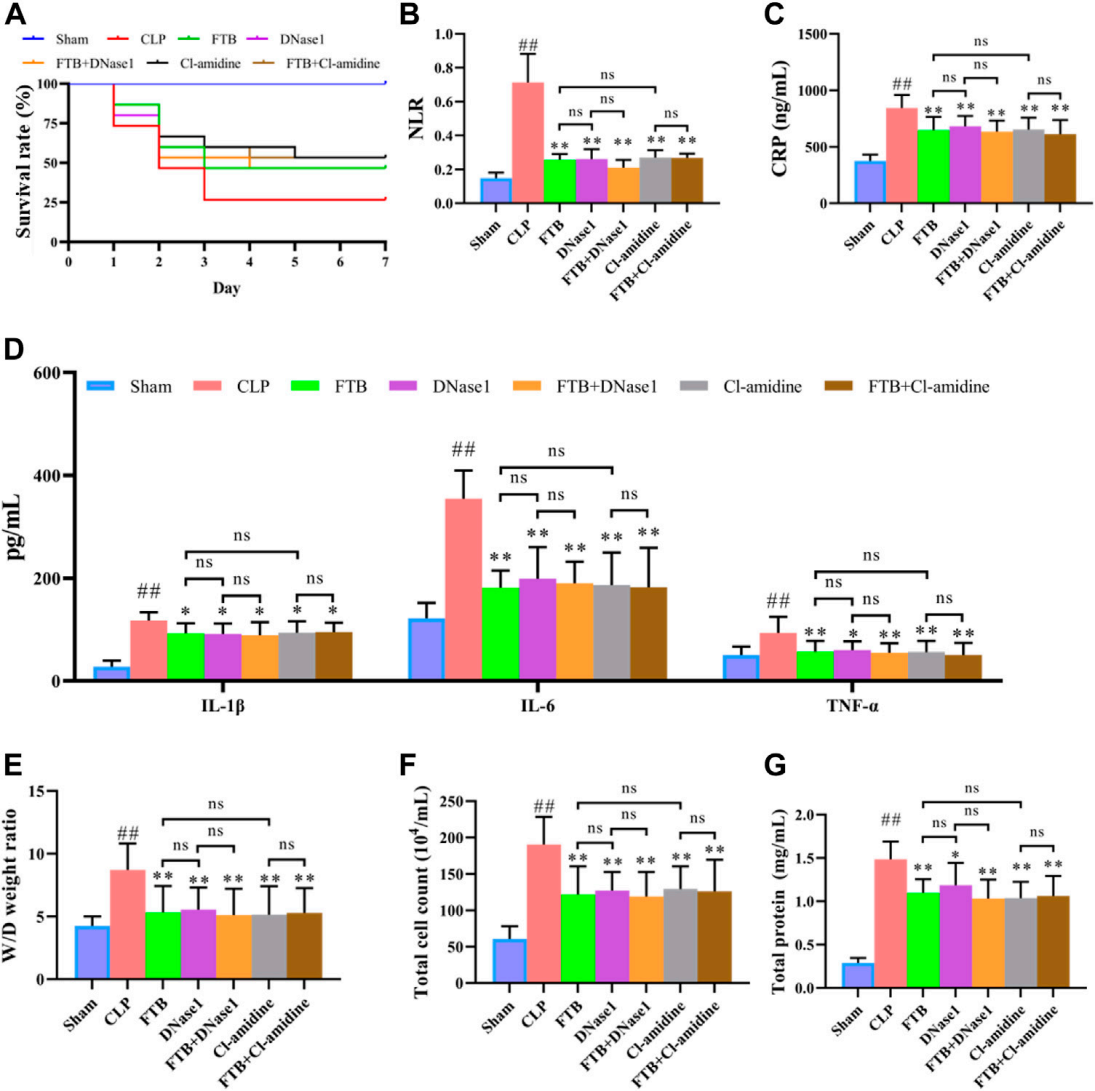
Effects of FTB treatment on survival rate, inflammatory response and lung permeability in rats with sepsis after inhibiting the formation of NET and the activity of PAD4. In rats with sepsis, treatment with FTB, DNase1 (NETs inhibitor) and Cl-amidine (PAD4 inhibitor) increased the survival rate **(A)**, reduced inflammation-related factors [NLR **(B)**, CLP **(C)** and pro-inflammatory cytokines **(D)**] and decreased lung permeability [W/D ratio of lung tissue **(E)**, total cell count **(F)** and total protein concentration **(G)** in BALF]. However, no differences were observed in the survival rate, inflammation-related factors and degree of lung permeability between the FTB and DNase1 groups, FTB and Cl-amidine groups, DNase1 and FTB + DNase1 groups, and Cl-amidine and FTB + Cl-amidine groups. Sham group (*n* = 15), CLP group (*n* = 8), FTB group (*n* = 10), DNase1 group (*n* = 9), FTB + DNase1 group (*n* = 10), Cl-amidine group (*n* = 10), FTB + Cl-amidine group (*n* = 10); ns: not statistically significant.

Subsequently, we used NETs inhibitors (DNase1) and PAD4 inhibitors (Cl-amidine) to further study the involvement of NETs and PAD4 in the anti-sepsis effect of FTB. After 7 days of treatment with FTB, the survival rate was 100% in the sham group and 26.7% in the CLP group. Treatment with FTB, DNase1, FTB + DNase1, Cl-amidine, or FTB + Cl-amidine increased the survival rate of rats with sepsis. The survival rates in the FTB, DNase1, FTB + DNase1, Cl-amidine, and FTB + Cl-amidine groups were 46.7%, 46.7%, 46.7%, 53.3%, and 53.3%, respectively (Figure 6A).

Additionally, the CLP group exhibited a higher NLR than the sham group. NLR was lower in the FTB, DNase1, FTB + DNase1, Cl-amidine, and FTB + Cl-amidine groups than in the CLP group (Figure 6B). ELISA results showed that the CLP group had significantly higher serum CRP, IL-6, IL-1β, and TNF-α levels compared with the sham group. FTB, DNase1, FTB + DNase1, Cl-amidine, and FTB + Cl-amidine treatment lowered the levels of CRP, IL-6, IL-1β, and TNF-α in rats with sepsis (Figures 6C,D).

The W/D ratio of the lung tissue, total protein concentration, and total cell count in BALF were higher in the CLP group than in the sham group. Compared with the rats in the CLP group, FTB, DNase1, FTB + DNase1, Cl-amidine, and FTB + Cl-amidine-treated rats showed lower W/D ratios of lung tissue, total protein concentration, and total cell count in BALF (Figures 6E–G). The assessment of serum indexes of the hepatic function showed that ALT and AST levels were significantly higher in the CLP group than in the sham group; FTB, DNase1, FTB + DNase1, Cl-amidine, and FTB + Cl-amidine groups had significantly lower ALT and AST levels compared with the CLP group (Table 3). Results of the renal function test showed that serum Cr and BUN levels were significantly higher in the CLP group than in the sham group; FTB, DNase1, FTB + DNase1, Cl-amidine, and FTB + Cl-amidine groups had significantly lower Cr and BUN levels compared with the CLP group (Table 3).

**TABLE 3:** Changes in ALT and AST activities, Cr and BUN levels after FTB, DNase1 and Cl-amidine treatment.

Group	ALT(U/L)	AST(U/L)	Cr(μmol/L)	BUN(mmol/L)
Sham	40.5 ± 13.0	103.7 ± 23.4	58.0 ± 14.1	4.4 ± 1.0
CLP	92.5 ± 22.1^##^	192.8 ± 27.1^##^	106.6 ± 20.3^##^	6.8 ± 1.6^##^
FTB	65.3 ± 13.5^**^	120.5 ± 47.5^**^	79.3 ± 23.2^*^	5.3 ± 1.4^*^
DNase1	67.3 ± 19.9^*^	123.1 ± 58.5^*^	76.3 ± 22.4^*^	5.5 ± 0.8^*^
FTB + DNase1	68.3 ± 20.7^*^	123.6 ± 68.3^*^	75.1 ± 12.9^**^	5.4 ± 0.6^*^
Cl-amidine	57.5 ± 20.8^**^	125.5 ± 27.9^**^	76.2 ± 19.2^**^	5.4 ± 1.2^*^
FTB + Cl-amidine	62.6 ± 21.7^*^	125.4 ± 61.9^*^	73.7 ± 23.1^**^	5.3 ± 1.2^*^

Sham group (*n* = 15), CLP group (*n* = 8), FTB group (*n* = 10), DNase1 group (*n* = 9), FTB + DNase1 group (*n* = 10), Cl-amidine group (*n* = 10), FTB + Cl-amidine group (n = 10).

**FIGURE 7:**
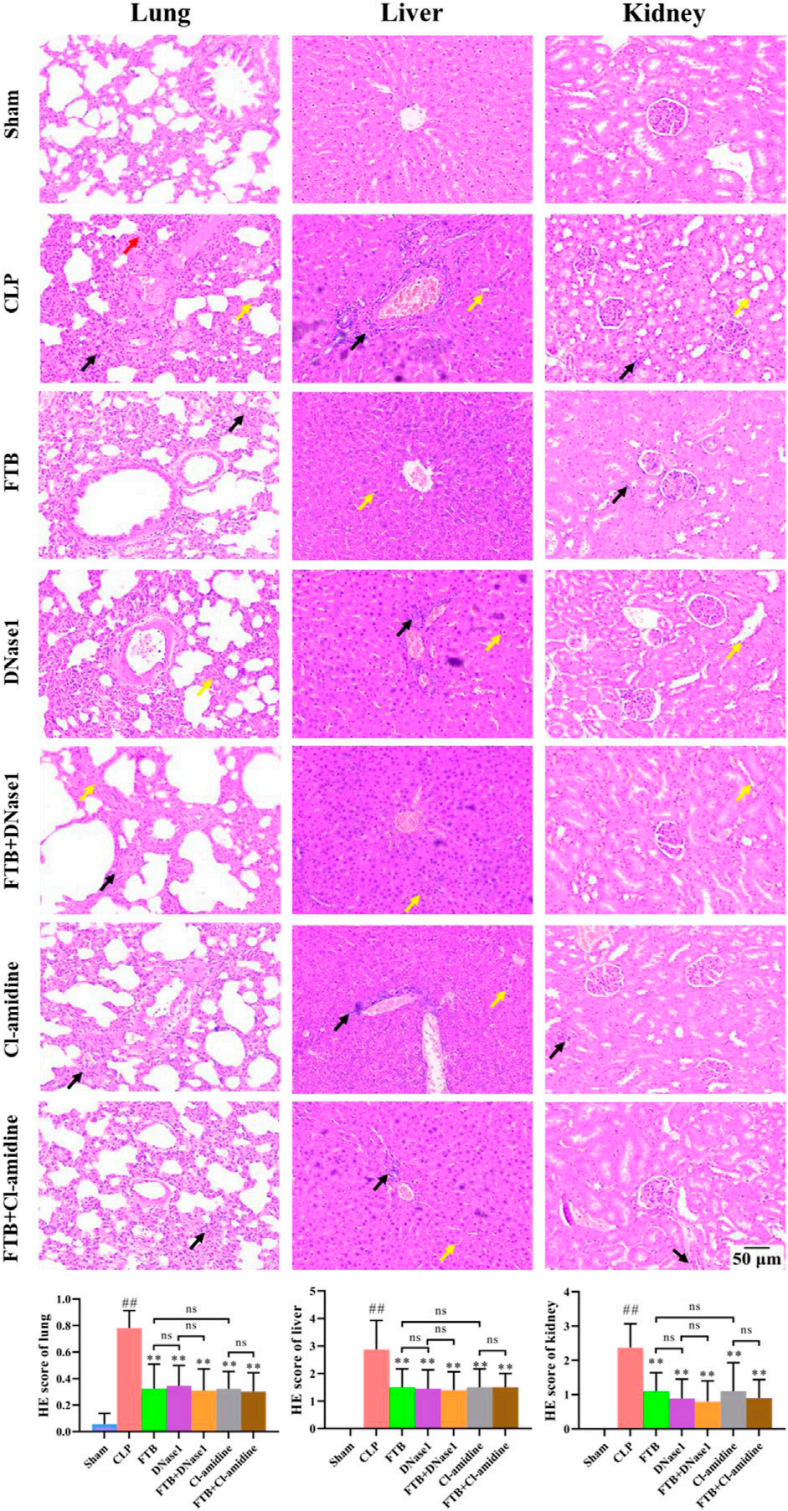
Effects of FTB on CLP-induced pathological changes and tissue injury scores of the lung, liver and kidney in rats with sepsis after inhibiting the NETs formation and the activity of PAD4. Treatment with FTB, DNase1 (NETs inhibitor) and Cl-amidine (PAD4 inhibitor) improved the CLP-induced pathological changes in the lung (red arrows indicate the pulmonary interstitial congestion, yellow arrows indicate the thickened alveolar walls and black arrows indicate the infiltration of inflammatory cells), liver (yellow arrows indicate the necrotic hepatocytes and black arrows indicate the infiltration of inflammatory cells) and kidney (yellow arrows indicate the enlarged lumen of focal tubules and black arrows indicate the interstitial inflammatory cell infiltration), and decreased the tissue injury scores. However, no differences were observed in the pathological changes and tissue injury scores between the FTB and DNase1 groups, FTB and Cl-amidine groups, DNase1 and FTB + DNase1 groups, and Cl-amidine and FTB + Cl-amidine groups (magnification: ×100). Sham group (*n* = 15), CLP group (*n* = 8), FTB group (*n* = 10), DNase1 group (*n* = 9), FTB + DNase1 group (*n* = 10), Cl-amidine group (*n* = 10), FTB + Cl-amidine group (*n* = 10).

Results of HE staining showed that FTB, DNase1, FTB + DNase1, Cl-amidine, and FTB + Cl-amidine treatments improved the pathological changes induced by CLP in the lungs, liver, and kidneys of rats. Additionally, the tissue injury scores of the lungs, liver, and kidneys were lower in the FTB, DNase1, FTB + DNase1, Cl-amidine, and FTB + Cl-amidine groups than in the CLP group (Figure 7).

Furthermore, no differences were observed in the levels of inflammatory factors, degree of lung injury, hepatic and renal functions, and histopathological changes between the FTB and DNase1, FTB and Cl-amidine, DNase1 and FTB + DNase1, and Cl-amidine and FTB + Cl-amidine groups.

### Effects of FTB on coagulation function in rats with sepsis after the inhibition of NETs formation and of PAD4 activity

**FIGURE 8:**
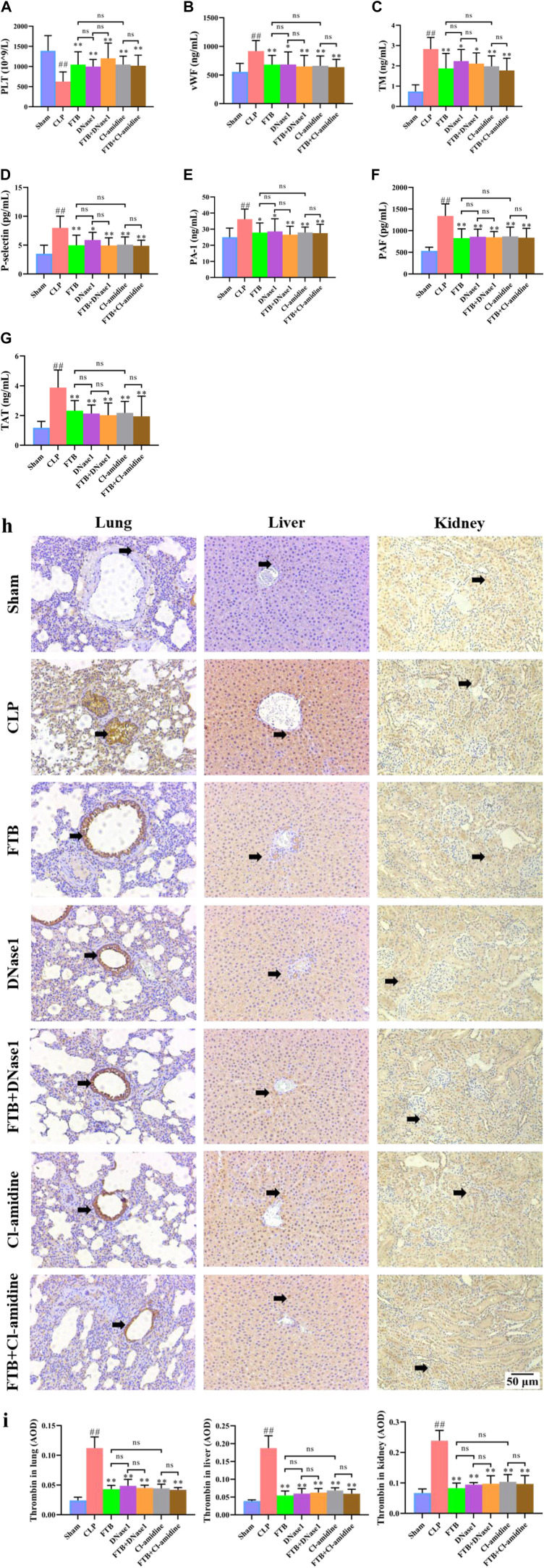
Effects of FTB on coagulation function in rats with sepsis after inhibiting the NET formation and PAD4 activity. In rats with sepsis, treatment with FTB, DNase1 (NETs inhibitor) and Cl-amidine (PAD4 inhibitor) increased the PLT count **(A)**, decreased the levels of coagulation factors [vWF **(B)**, TM **(C)**, P-selectin **(D)**, PAI-1 **(E)**, PAF **(F)**, TAT **(G)**] and lowered the expression of thrombin in lung, liver and kidney (black arrows indicate the positive expression areas) **(H,I)**. However, no differences were observed in coagulation function between the FTB and DNase1 groups, FTB and Cl-amidine groups, DNase1 and FTB + DNase1 groups, and Cl-amidine and FTB + Cl-amidine groups (magnification: ×100). Sham group (*n* = 15), CLP group (*n* = 8), FTB group (*n* = 10), DNase1 group (*n* = 9), FTB + DNase1 group (*n* = 10), Cl-amidine group (*n* = 10), FTB + Cl-amidine group (*n* = 10).

Compared with the sham group, the CLP group had a significantly lower PLT count in the peripheral blood; compared with the CLP group, the FTB, DNase1, FTB + DNase1, Cl-amidine, and FTB + Cl-amidine groups had significantly higher PLT count (Figure 8A). Compared with the sham group, the CLP group had significantly longer APTT and PT and a significantly lower FIB level; compared with the CLP group, the FTB, DNase1, FTB + DNase1, Cl-amidine, and FTB + Cl-amidine groups had shorter APTT and PT levels and a significantly higher FIB level (Table 4).

**TABLE 4:** Changes in APTT, PT, TT and FIB levels after FTB, DNase1 and Cl-amidine treatment.

Group	APTT(s)	PT(s)	TT(s)	FIB(μg/L)
Sham	19.7 ± 4.7	20.7 ± 6.8	22.4 ± 4.2	3.1 ± 1.0
CLP	40.9 ± 14.1^##^	47.1 ± 8.7^##^	19.7 ± 5.0	1.7 ± 0.2^##^
FTB	28.3 ± 6.7^*^	29.1 ± 6.2^**^	25.3 ± 7.9	3.1 ± 0.6^**^
DNase1	28.6 ± 3.7^*^	34.0 ± 6.2^**^	23.3 ± 5.2	2.7 ± 0.7^**^
FTB + DNase1	27.5 ± 4.0^*^	31.1 ± 2.6^**^	24.5 ± 4.3	2.7 ± 0.8^**^
Cl-amidine	23.1 ± 7.8^**^	36.4 ± 7.8^*^	24.3 ± 6.2	2.8 ± 0.5^**^
FTB + Cl-amidine	23.4 ± 6.3^**^	38.9 ± 6.4^*^	24.3 ± 7.7	2.7 ± 0.6^**^

Sham group (*n* = 15), CLP group (*n* = 8), FTB group (*n* = 10), DNase1 group (*n* = 9), FTB + DNase1 group (*n* = 10), Cl-amidine group (*n* = 10), FTB + Cl-amidine group (*n* = 10).

The ELISA results indicated that the levels of coagulation factors (vWF, TM, P-selectin, PAI-1, PAF, and TAT) were significantly higher in the CLP group compared with the sham group. Compared with the CLP group, the FTB, DNase1, FTB + DNase1, Cl-amidine, and FTB + Cl-amidine groups showed lower levels of serum vWF, TM, P-selectin, PAI-1, PAF, and TAT (Figures 8B–G).

IHC results showed that the thrombin-positive expression area in the lung, liver, and kidney tissues was significantly larger in the CLP group than in the sham group. Compared with the CLP group, the FTB, DNase1, FTB + DNase1, Cl-amidine, and FTB + Cl-amidine groups had smaller thrombin-positive expression areas in the lungs, liver, and kidneys (Figures 8H,I).

Moreover, no significant differences were observed in the levels of PLT, APTT, PT, FIB, vWF, TM, P-selectin, PAI-1, PAF, and TAT and thrombin expression in the lungs, liver, and kidneys between the FTB and DNase1 groups, FTB and Cl-amidine groups, DNase1 and FTB + DNase1 groups, and Cl-amidine and FTB + Cl-amidine groups.

## Discussion

In this study, the CLP method was used to establish a rat model of sepsis. The CLP model is recognized as one of the most utilized sepsis models because it is characterized by a pathological process similar to sepsis in humans (Dejager et al., 2011). Our results demonstrated that the survival rate of rats that received CLP was less than 40%, indicating that the model used in our study was successful (Silva et al., 2010). Three dosages (20, 40, and 80 mg/kg) of FTB were used to treat model rats with sepsis. A previous study showed that the intravenous injection of FTB (20 and 40 mg/kg) could reduce the inflammatory response in rats with CLP-induced sepsis (Jiang et al., 2013). Moreover, intraperitoneal administration of 50 and 100 mg/kg FTB ameliorated LPS-induced acute lung injury in mice (Liu et al., 2019). Our results showed that treatment with 40 and 80 mg/kg FTB increased the survival rate of rats with sepsis. The pathological basis of sepsis is a systemic inflammatory response syndrome caused by an infection. Therefore, the effective control of systemic inflammation is an efficacious measure to reduce sepsis-related mortality (Jianjun et al., 2018). The levels of inflammatory factors in rats were significantly elevated 6–48 h post-CLP induction (Zafrani et al., 2012; Jia et al., 2021). Our results also revealed that 24 h after ligation, serum levels of CRP, IL-6, IL-1β, and TNF-α were significantly higher in CLP rats than in sham controls, suggesting that CLP induces a severe systemic inflammatory response. However, FTB intervention reduced the serum levels of these proinflammatory factors in rats with sepsis. FTB also decreased NLR, which is an important indicator of sepsis-associated inflammation. Furthermore, multiple organs successively fail, and hepatic and renal functions are seriously damaged 24 h after the onset of sepsis (C.-M. Jia et al., 2021; Zafrani et al., 2012). Our results showed that serum biochemical parameters related to hepatic and renal function were significantly elevated in the rats with sepsis compared with sham control, whereas FTB improved these functions. HE staining revealed that FTB significantly improved pathological changes of the liver and kidneys of rats with sepsis. Lung injury is an important pathological change in sepsis (David et al., 2015). We tested the W/D ratio of the lung tissue, total cell counts, and total protein content in the BALF. These are important indicators in evaluating lung injury, and the increase in the W/D ratio of lung tissue, total cell counts and total protein content in BALF can reflect the increase in lung permeability (Jia et al., 2022). Lung permeability increases during lung injury. Our staining revealed significant lung injury in the rats following CLP induction. FTB treatment decreased lung permeability and ameliorated lung pathological changes induced by CLP. These results indicate FTB has a significant therapeutic effect on sepsis.

The onset of sepsis was associated with varying degrees of coagulopathies (Zeerleder et al., 2005; Schouten et al., 2007; Levi & Poll, 2015). The results of routine blood and coagulation function tests showed that PLT and FIB levels were lower and that APTT and PT were longer in rats with sepsis than in sham controls. Thrombocytopenia is a frequent multifactorial phenomenon that occurs during sepsis. Decreased PLT production is accompanied by increased PLT consumption and destruction (Vardon-Bounes et al., 2019). Sepsis can lead to coagulation disorders, and FIB levels decrease over time (Mori et al., 2022). Additionally, decreased FIB was closely associated with mortality; the mortality sharply increased when the level of FIB was <200 mg/dl and antithrombin activity was <50% (Matsubara et al., 2019). Patients with sepsis showed increased levels of PT and APTT (Huan et al., 2013). Our results also demonstrated that FTB increased the levels of PLT and FIB and shortened the APTT and PT in rats with sepsis. Moreover, the effects of FTB on the levels of coagulation-related factors vWF, TM, P-selectin, PAI-1, PAF, and TAT in rats with sepsis were assessed. The results showed that FTB significantly reduced the levels of vWF, TM, P-selectin, PAI-1, PAF, and TAT in the peripheral blood compared with CLP procedure only. Both vWF and TM are markers of endothelial cell activation and their levels are elevated during sepsis (López-Aguirre and Páramo, 1999). Additionally, P-selectin levels are elevated in patients with sepsis and that is related to increased P-selectin levels in individual PLT and activated endothelial cells (Fijnheer et al., 1997). PAI-1 is the main inhibitor of fibrinolysis in sepsis (Zeerleder et al., 2006). Patients with sepsis generally have high PAI-1 levels, which are considered to be associated with disease severity (Raaphorst et al., 2001). PAF is a bioactive phospholipid that induces PLT aggregation, thereby causing thrombosis (M et al., 1979). TAT, a combination of thrombin and antithrombin, is a sensitive marker that activates the coagulation system and directly reflects the level of thrombin generation (Pelzer, 1989). IHC results revealed that treatment with FTB reduced thrombin expression in lung, liver, and kidney tissues.

NETs are three-dimensional reticular structures, with DNA as the backbone to which various protein components bind. Their excessive production can activate the coagulation system, inhibit the anticoagulation system, resist fibrinolysis, damage vascular endothelial cells, all these phenomena resulting in sepsis-induced coagulopathy *via* PLT interactions (Denning et al., 2019). To explore the effects of FTB on NET levels in the peripheral blood of rats with sepsis, we first extracted neutrophils from the peripheral blood of rats in each group, induced NET production with PMA, and stained the cells with immunofluorescent anti-MPO, anti-H3-Cit, and SYTOX Green. PMA is a protein kinase C (PKC) activator that can be used to promote NET production in human and rat neutrophils (Nakazawa et al., 2012; Uozumi et al., 2019). MPO is a unique neutrophil marker. Neutrophil MPO is directly related to the number of neutrophils required for their identification (Bradley et al., 1982). Our results showed that all the cells expressed MPO. Both Cit H3 and SYTOX Green staining have been used to detect NETs (Masuda et al., 2017; Mauracher et al., 2018). We revealed that levels of NETs in CLP rats were significantly elevated compared with sham controls, and FTB inhibited NET production in these rats. Furthermore, we also evaluated the effects of FTB on PAD4 expression in the neutrophils of rats with sepsis. PAD is a family of enzymes involved in post-translational modification. They catalyze the conversion of positively charged protein-bound arginine and methylarginine residues to the uncharged, non-standard amino acid citrulline, which affects substantially biochemical pathways (Vossenaar et al., 2003; Denman, 2005). PAD4 is a subtype of enzyme belonging to this family and is expressed in granulocytes. Histone citrullination mediated by PAD4 leads to histone hypercalcification and chromatin decondensation, which are key factors in NETs formation. Indeed, PAD4^−/−^ neutrophils cannot form NETs after stimulation with chemokines or incubation with bacteria and are deficient in NETs-induced bacterial killing (Rohrbach et al., 2012). Our results showed that FTB significantly reduced PAD4 expression in neutrophils, suggesting that it inhibited NET formation and improved coagulopathies in rats with sepsis by reducing PAD4 expression in neutrophils.

To further confirm the mechanism by which FTB mitigates sepsis-associated coagulopathies, we used the NETs inhibitor DNase1 and the PAD4 inhibitor Cl-amidine. DNase1 is a frequently used inhibitor of NETs. An injection of 5 mg/kg DNase1 into the caudal vein of rats significantly inhibited NETs formation (Weber et al., 2019). Cl-amidine is a frequently used PAD4 inhibitor. In rats, an intraperitoneal injection of 50 mg/kg Cl-amidine significantly reduced PAD4 expression and inhibited NETs formation (Feng et al., 2021). These results imply that both NETs and PAD4 inhibitors have a certain therapeutic effect on sepsis and could improve the associated coagulopathies, which is consistent with previously reported results (Biron et al., 2016; Zhao et al., 2016; Jiménez-Alcázar et al., 2017). The therapeutic effects of FTB on sepsis and associated coagulopathy were similar to those of the monotherapy with NETs and PAD4 inhibitors; compared with monotherapy with NETs and PAD4 inhibitors, the association of FTB with NETs or PAD4 inhibitors did not enhance the therapeutic effect. However, we did not test the NET formation and PAD4 expression in study using NET and PAD4 inhibitors. Further studies should be conducted to address this limitation.

In conclusion, our study confirmed that FTB has a therapeutic effect in rats with sepsis and alleviates associated coagulopathies. Furthermore, these effects of FTB result from the prevention of NETs formation by reduction of PAD4 expression.

## Data Availability

The original contributions presented in the study are included in the article/supplementary material further inquiries can be directed to the corresponding author.
